# What Is Your Diagnosis? Abnormal WDF Scattergram From the Sysmex XN‐1000V in a Cat

**DOI:** 10.1111/vcp.70030

**Published:** 2025-09-19

**Authors:** Javier Martínez‐Caro, Laura Chaves‐Falcón, Marta Lemos, Xavier Roura, Luis Bosch, Josep Pastor

**Affiliations:** ^1^ Servei d’Hematologia Clínica Veterinària, Departament de Medicina i Cirurgia Animals, Facultat de Veterinària Universitat Autònoma de Barcelona Bellaterra Spain; ^2^ Hospital Clínic Veterinari Universitat Autònoma de Barcelona Bellaterra Spain

**Keywords:** feline, fibrin, hematology, leukocytes, WDF scattergram

## 1 Case Presentation

A 2‐year‐old, neutered male, domestic shorthair cat was readmitted to our center for worsening of a feline respiratory disease complex. It was first presented 11 days earlier (Day 1) with a 1‐week history of complaints of coughing, sneezing, abundant mucus production, open‐mouth breathing, apathy, and anorexia. The cat was neither vaccinated nor dewormed. A FeLV/FIV screening test (SNAP Combo, IDEXX Laboratories) was performed with negative results. After 6 days of hospitalization with supportive care, the cat showed a good recovery and was discharged with oral medication consisting of doxycycline (5 mg/kg q12h), buprenorphine (0.015 mg/kg q8h), prednisolone (1.5 mg/kg q24h), nucleotides + AHCC (Impromune, ½ tablet q24h) and esophagostomy tube feeding. On readmission (Day 12), the physical examination revealed pink mucous membranes with mild periodontal secretion and gingival ulcers, adequate capillary refill time, weak pulse, rectal temperature of 35.5°C, and depressed mental status.

On Days 1, 6, and 12, CBC and plasma biochemistry were performed on a ProCyte Dx and Catalyst DX (IDEXX, Westbrook, USA), respectively (Tables S1 and S2, Figure S1). The blood smears were not reviewed on Days 1 and 6. The CBC of Day 12 was repeated using the Sysmex XN‐1000V (Sysmex Corporation, Norderstedt, Germany). Results are presented in Table S1 and WDF scattergrams in Figure 1.

**FIGURE 1:**
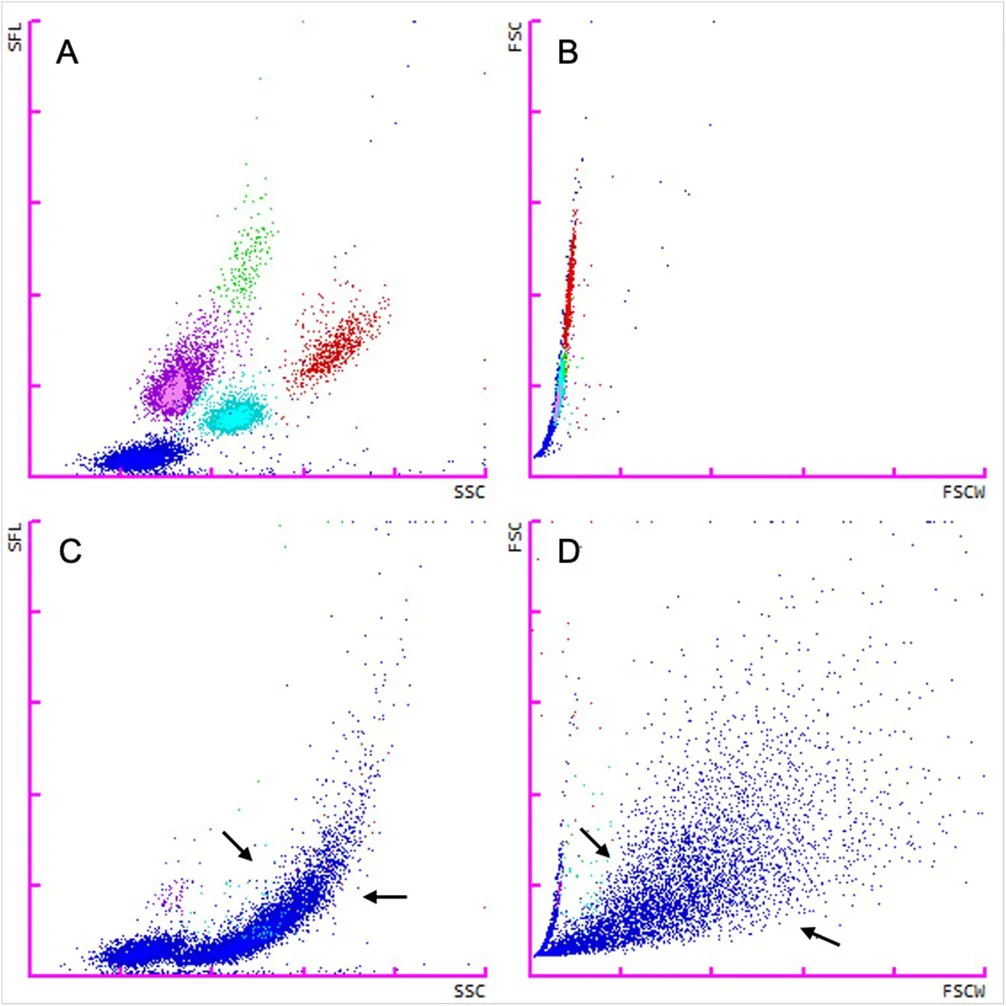
Feline peripheral blood scattergrams from the WDF channel from the Sysmex XN‐1000V. (A, B) show an example of a healthy cat. (C, D) show the blood of the cat from this report on Day 12. The arrows indicate the abnormal clusters. FSC, forward scattered light intensity; FSCW, forward scattered light pulse width; SFL, side fluorescence light intensity; SSC, side scattered light intensity. Particle representation: Cyan dots (neutrophils), purple dots (lymphocytes), green dots (monocytes), red dots (eosinophils), and dark blue dots (debris).

### 1.1 Scattergram Interpretation

Panleukopenia with an abnormal curvilinear cluster in the right middle‐lower region of the WDF scattergram of a likely interfering substance. Considerations included platelet clumps, but also cryoglobulin precipitates, fibrin strands, or others, such as ultrasound gel, hyaluronic acid influence, or other unknown interfering substances.

To rule out a spurious result due to analyzer failure, the analysis was repeated in the Sysmex hematology analyzer, confirming the previous CBC results and the abnormal WDF cluster (data not shown). The sample was also analyzed in the Pre‐diluted (PD) mode, using 20 μL of blood and 120 μL of CELLPACK DCL solution (Sysmex Corporation, Norderstedt, Germany), yielding similar CBC results (data not shown) and almost complete disappearance of the abnormal WDF cluster (Figure S2). In this case, the WNR scattergrams showed no interference or additional clusters in the initial or any of the above analyses. Blood smears were performed, air dried, and stained on a Hematek 2000 (Siemens Healthcare Diagnostics, Deerfield, IL, USA) using a Wright‐Giemsa stain (Hematek Stain Pak—Wright‐Giemsa stain, Siemens Healthcare Diagnostics, Deerfield, IL, USA). Peripheral blood smear examination (Figure 2) revealed abundant extracellular proteinaceous material, elongated to fibrillar, light blue to purple, morphologically interpreted as fibrin deposits (fibrin strands and clumps). This material was distributed throughout the whole smears, but was more abundant in the feathered edge. The marked leukopenia was confirmed on the blood smear with scattered WBC (< 5 WBC per blood smear), mostly small lymphocytes and fewer monocytes and neutrophils (either segmented and bands). No significant toxic change in neutrophils was observed. Red blood cells did not show significant poikilocytosis but just mild crenation. The platelets were manually estimated as 110–134 thousand per microliter, with very scant platelets trapped in the fibrin aggregates, but not significant platelet clumping.

**FIGURE 2:**
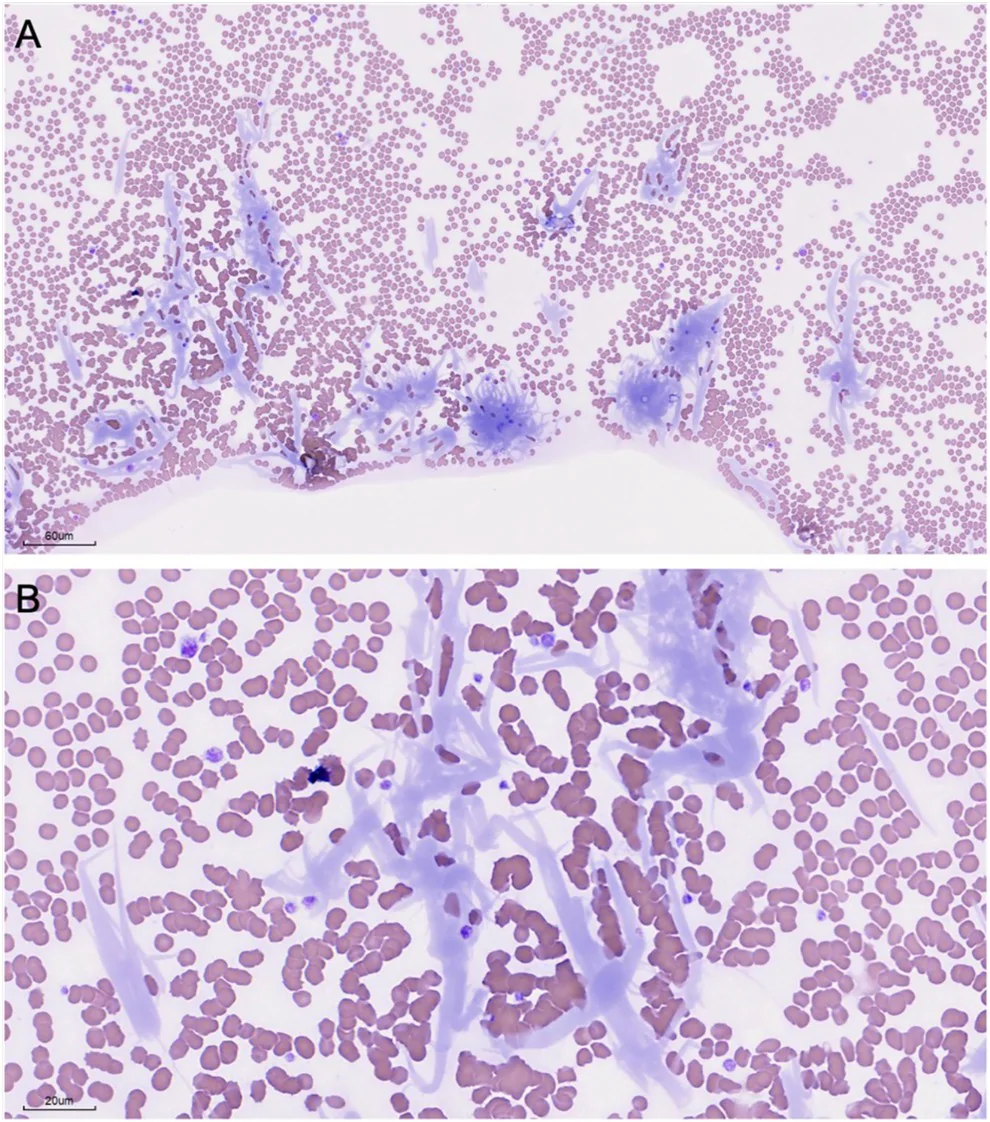
Peripheral blood smear micrographs from a 2‐year‐old, neutered male, domestic shorthair cat (Day 12). Notice abundant extracellular proteinaceous material, elongated to fibrillar, light blue to purple, interpreted as fibrin deposits. Wright‐Giemsa stain, images acquired using a digital slide scanner (Motic EasyScan One, MoticEurope SLU, Barcelona, Spain). Image (A, B) at ×20 and ×40 magnification, respectively.

### 1.2 Additional Results

Based on the WDF scattergram morphology and the previous descriptions of fibrillar to needle‐like cryoglobulin precipitates [1], cryoglobulinemia remained in the differential diagnosis. To test this hypothesis, the sample was incubated at 37°C for 30 min, and new blood smears were immediately prepared. Microscopic evaluation of the new blood smears revealed similar morphology and amount of the protein precipitates, which were again interpreted as fibrin deposits.

Special staining to confirm the fibrin nature of the proteinaceous deposits was performed on Wright‐Giemsa‐stained slides, after destaining by immersion in methanol for 6 h. Mallory's Phosphotungstic Acid Hematoxylin (PTAH) stain was prepared in‐house according to previously published protocols [2]. Two positive controls were used, one of which was Wright‐Giemsa‐stained and methanol‐destained, to exclude Wright‐Giemsa effects on the PTAH staining. The fibrin nature of the precipitates was confirmed by the blue staining of the fibrillar, extracellular, proteinaceous material (Figure 3).

**FIGURE 3:**
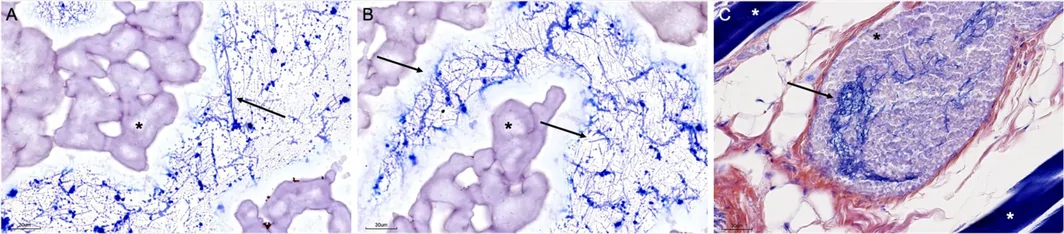
Mallory's Phosphotungstic Acid Hematoxylin staining micrographs. (A, B) Peripheral blood smear from a 2‐year‐old, neutered male, domestic shorthair cat (Day 12); notice that the abundant, extracellular, fibrillar, proteinaceous material, which is stained dark blue and interpreted as fibrin (arrows). (C) Skeletal muscle tissue, positive control; notice that the fibrin meshwork (arrow) and skeletal muscle fibers (white asterisks) are stained dark blue, and collagen fibers are stained orange to red. Red blood cells are stained brown (black asterisks). Mallory's Phosphotungstic Acid Hematoxylin stain, images acquired using a digital slide scanner (Motic EasyScan One, MoticEurope SLU, Barcelona, Spain). Image A, B and C at ×40 magnification.

Shortly after readmission on Day 12, the cat died without further tests; the owners declined to perform a necropsy.

## 2 Discussion

The most clinically relevant hematological finding was the marked leukopenia. Causes of neutropenia include decreased release of neutrophils from the bone marrow, increased migration of neutrophils from the blood to peripheral tissues, destruction, or shift of neutrophils from the circulating to the marginating neutrophil pool [3]. Given the clinical signs, overwhelming inflammation was strongly suspected; possible causes include endotoxemia or sepsis [4]. Another possibility could be a parvoviral infection, but feline panleukopenia virus infection was not further investigated. The severe panleukopenia observed on both analyzers correlated with the degree of leukopenia observed in the blood smear review and was judged to be real. However, the question of how much contribution could be expected from the WBC being trapped by the fibrin aggregates remains unclear.

The mild thrombocytopenia may be artifactual if some degree of platelet clumping occurred inadvertently in vitro or may be due to increased platelet utilization. Other causes include increased platelet destruction, decreased bone marrow production, sequestration, and blood loss (which is unlikely due to a normal erythron) [5].

The presence of visible fibrin strands on the blood smears is often attributed to in vitro formation due to the partial clotting of the blood sample. However, fibrin strands can also be seen in patients with a hypercoagulable state [6, 7, 8]. In this cat, the prominent amount of fibrin and the absence of visible platelet aggregation led us to suspect that some in vivo fibrin formation might be happening. A hypercoagulable state in this cat would fit with the clinical signs suggestive of septicemic shock; however, no additional tests were performed to confirm microvascular thrombosis and/or disseminated intravascular coagulation [9].

In the WBC scattergram from the Procyte DX (Figure S1), the atypical cluster was not as prominent as in the WDF scattergram from the Sysmex XN‐1000V. This may be due to technical differences between the analyzers or additional in vitro fibrin formation. Analysis of a new sample taken at that time point would have been beneficial.

In conventional flow cytometry, a forward scatter height versus forward scatter area plot is commonly used to identify and exclude doublets. Similarly, the Sysmex XN‐1000V WDF channel displays a forward scattered light intensity versus forward scattered light pulse width (FSCW) plot. This scattergram is used to identify some interferents, such as platelet clumps (Figure 4C,D), cryoglobulins (Figure 4G,H), and fibrin (Figure 1), which have higher FSCW than single cells. Apparently, similar WDF scattergrams are observed for cases with abundant platelet clumps and cases with abundant fibrin deposits, so it is not possible to elucidate how much platelets or fibrin contribute to this scattergram in those cases where both are present.

**FIGURE 4:**
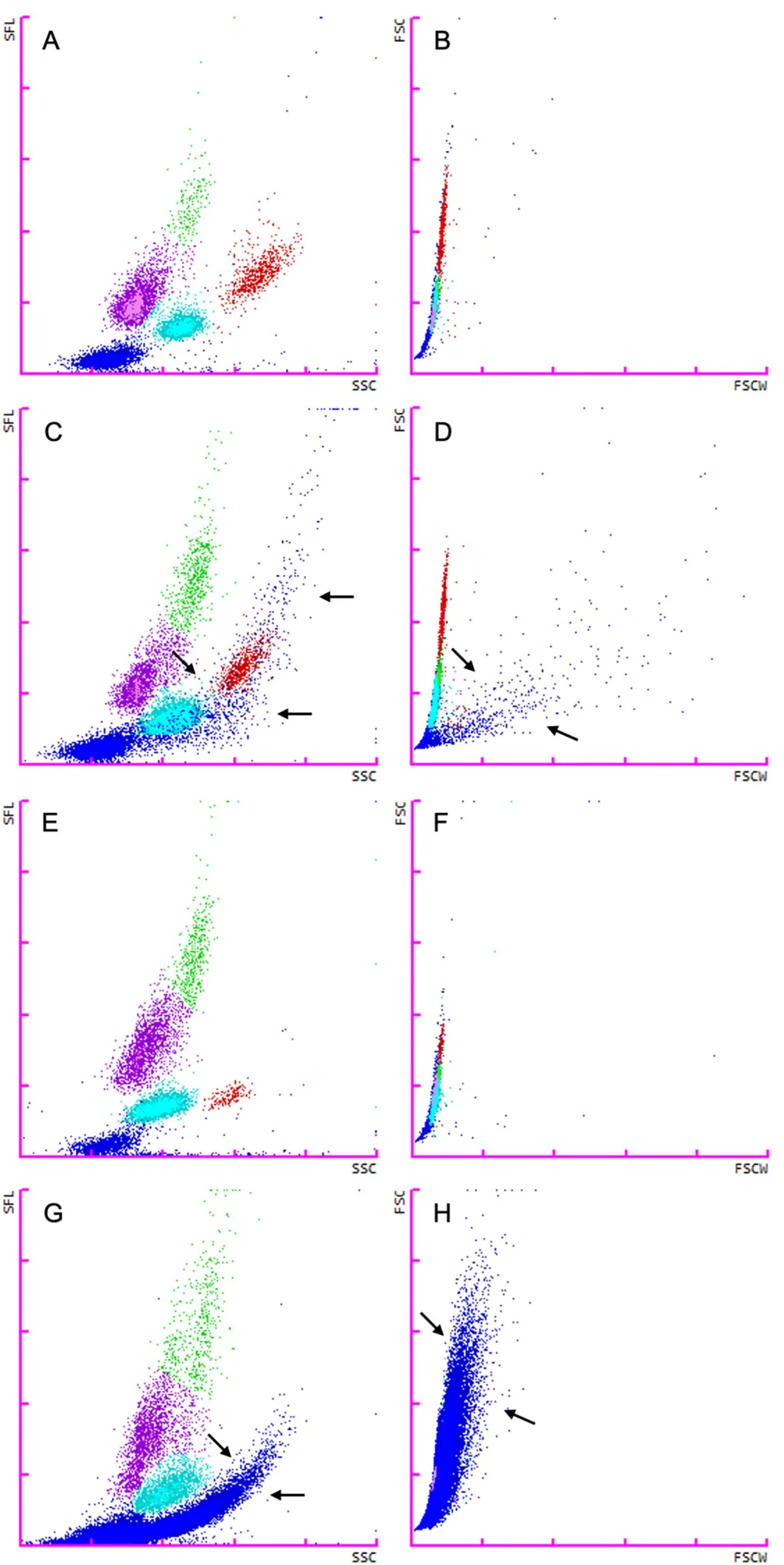
Feline (A–D) and canine (E–H) peripheral blood scattergrams from the WDF channel from the Sysmex XN‐1000V. (A, B) show an example of a healthy cat. (C and D) show an example of a cat with prominent platelet aggregation without visible fibrin strands on the blood smear. (E, F) show an example of a healthy dog. (G, H) show an example of a dog with confirmed cryoglobulinemia. The arrows indicate the abnormal clusters. FSC, forward scattered light intensity; FSCW, forward scattered light pulse width; SFL, side fluorescence light intensity; SSC, side scattered light intensity. Particle representation: Cyan dots (neutrophils), purple dots (lymphocytes), green dots (monocytes), red dots (eosinophils), and dark blue dots (debris).

In conclusion, to the authors' knowledge, this is the first human or veterinary peer‐reviewed report of fibrin strands and clumps alone, without significant platelet aggregation, associated with an atypical cluster on the WDF scattergrams from the Sysmex XN‐1000V. Visualization of fibrin on peripheral blood smears may indicate preanalytical errors, but may also reflect some clinically relevant conditions such as thrombosis or other coagulation disorders. Scattergram and blood smear evaluation are essential tools for providing accurate hematology results.

## Conflicts of Interest

The authors declare no conflicts of interest.

## Supporting information


**Data S1:** vcp70030‐sup‐0001‐DataS1.zip.
**Figure S1:** Feline peripheral blood scattergrams from the WBC channel from the ProCyte Dx. (A) shows an example of a healthy cat. (B–D) show the blood of the cat from this report on Days 1, 6, and 12, respectively. SFL, side fluorescence light intensity; SSC, side scattered light intensity. Particle representation: purple (neutrophils), dark blue dots (lymphocytes), red dots (monocytes), green dots (eosinophils), cyan dots (basophils), and orange dots (debris).
**Figure S2:** Feline peripheral blood scattergrams from the WDF channel from the Sysmex XN‐1000V. A and B show an example of a healthy cat. (C and D) show the blood of the cat from this report on Day 12, analyzed using the Pre‐diluted (PD) mode. Note the almost complete disappearance of the abnormal WDF cluster. The arrows indicate the position where the abnormal cluster previously was. FSC, forward scattered light intensity; FSCW, forward scattered light pulse width; SFL, side fluorescence light intensity; SSC, side scattered light intensity. Particle representation: cyan dots (neutrophils), purple dots (lymphocytes), green dots (monocytes), red dots (eosinophils), and dark blue dots (debris).
**Table S1:** Hematology results for EDTA‐blood specimens performed on Days 1, 6, and 12, from a 2‐year‐old, domestic shorthair cat. Bolded values are outside the RI. HGB, hemoglobin; HCT, hematocrit; MCH, mean corpuscular hemoglobin; MCHC, mean corpuscular hemoglobin concentration; MCV, mean cell volume; MPV, mean platelet volume; PLT, platelets; PCT, plateletcrit; RBC, red blood cells; RET, reticulocytes; RET‐HE, reticulocyte hemoglobin equivalent; RI, reference interval; RDW, red cell distribution width; WBC, white blood cells; *, estimated adequate based on blood smear evaluation.
**Table S2:** Biochemistry results for heparin‐plasma specimens performed on Days 1, 6 and 12, from a 2‐year‐old, domestic shorthair cat. Bolded values are outside the RI. ALP, alkaline phosphatase; ALT, alanine aminotransferase; BUN, blood urea nitrogen; GGT, γ‐glutamyl transferase; RI, reference interval.
