# Supplementary material for: What Is Your Diagnosis? Abnormal WDF Scattergram From the Sysmex XN‐1000V in a Cat
Source: Vet Clin Pathol. 2025 Sep 19;55(Suppl 1):S41–5. doi: 10.1111/vcp.70030 (PMC13666550; doi:10.1111/vcp.70030)
Supplement: Supplementary file 1 [file VCP-55-S41-s001.zip › vcp70030-sup-0001-Supplementary_figure_1-2.docx]

FIGURE S1: Feline peripheral blood scattergrams from the WBC channel from the ProCyte Dx. A, shows an example of a healthy cat. B, C and D, show the blood of the cat from this report on days 1, 6, and 12, respectively. Abbreviations: SFL, side fluorescence light intensity; SSC, side scattered light intensity. Particle representation: purple (neutrophils), dark blue dots (lymphocytes), red dots (monocytes), green dots (eosinophils), cyan dots (basophils), and orange dots (debris).

FIGURE S2: Feline peripheral blood scattergrams from the WDF channel from the Sysmex XN-1000V. A and B show an example of a healthy cat. C and D show the blood of the cat from this report on day 12, analyzed using the Pre-diluted (PD) mode. Note the almost complete disappearance of the abnormal WDF cluster. The arrows indicate the position where the abnormal cluster previously was. Abbreviations: FSC, forward scattered light intensity; FSCW, forward scattered light pulse width; SFL, side fluorescence light intensity; SSC, side scattered light intensity. Particle representation: cyan dots (neutrophils), purple dots (lymphocytes), green dots (monocytes), red dots (eosinophils), and dark blue dots (debris).
