# Supplementary material for: What Is Your Diagnosis? Abnormal WDF Scattergram From the Sysmex XN‐1000V in a Cat
Source: Vet Clin Pathol. 2025 Sep 19;55(Suppl 1):S41–5. doi: 10.1111/vcp.70030 (PMC13666550; doi:10.1111/vcp.70030)
Supplement: Supplementary file 1 [file VCP-55-S41-s001.zip › vcp70030-sup-0002-Supplementary_tables_1-2.docx]

Table S1

| Variable | ProCyte Dx | | | | Sysmex XN-1000V | |
| --- | --- | --- | --- | --- | --- | --- |
|  | Result day 1 | Result day 6 | Result day 12 | RI | Result day 12 | RI |
| RBC (x10^12^/L) | 8.78 | 7.32 | 7.52 | 6.54-12.20 | 7.58 | 6-10.2 |
| HCT (%) | 38.2 | **29.9** | 31 | 30.3-52.3 | 29.0 | 27-48 |
| HGB (g/dL) | 12.2 | 10.1 | 10.3 | 9.8-16.2 | 10.5 | 9-15 |
| MCV (fL) | 43.5 | 40.8 | 41.2 | 35.9-53.1 | **38.3** | 41-53 |
| MCH (pg) | 13.9 | 13.8 | 13.7 | 11.8-17.3 | 13.9 | 13-17 |
| MCHC (g/dL) | 31.9 | 33.8 | 33.2 | 28.1-35.8 | **36.2** | 30-34 |
| RDW (%) | 24.3 | 23.9 | 24 | 15.0-27.0 | 18.4 | --- |
| RET (x10^9^/L) | 7 | 8.1 | **2.3** | 3-50 | 4.5 | 0-50 |
| RET-He (pg) | 15 | 14.6 | 14.6 | 13.2-20.8 | 14.8 | 13.7-19.3 |
| WBC (x10^9^/L) | 10.89 | 13.46 | **0.2** | 2.87-17.02 | **0.08** | 5-15 |
| Neutrophils (x10^9^/L) | 4.16 | 9.06 | **0.08** | 2.30-10.29 | --- | 2.5-11.3 |
| Lymphocytes (x10^9^/L) | 5.52 | 2.56 | **0.09** | 0.92-6.88 | --- | 1.4-6.1 |
| Monocytes (x10^9^/L) | 0.46 | 0.49 | **0.01** | 0.05-0.67 | --- | 0.1-0.6 |
| Eosinophils (x10^9^/L) | 0.38 | 0.88 | **0.00** | 0.17-1.57 | --- | 0-1.5 |
| Basophils (x10^9^/L) | --- | **0.47** | --- | 0.01-0.26 | --- | 0-0.1 |
| PLT (x10^9^/L) | **103*** | **133*** | **103** | 151-600 | **113** | 200-600 |
| MPV (fL) | 15 | 14.6 | 16.1 | 11.4-21-6 | 10.4 | --- |
| PCT (%) | **0.15** | 0.19 | 0.17 | 0.17-0.86 | 0.04 | --- |

Table S2

| Variable | Catalyst DX | | | |
| --- | --- | --- | --- | --- |
|  | Result day 1 | Result day 6 | Result day 12 | RI |
| Glucose (mg/dL) | **160** | --- | 118 | 74-159 |
| Creatinine (mg/dL) | 1.1 | 1.3 | 1.8 | 0.8-2.4 |
| BUN (mg/dL) | 22 | --- | 18 | 16-36 |
| Urea/creatinine ratio | 20 | --- | 10 | --- |
| Phosphate (mg/dL) | **7.6** | --- | --- | 3.1-7.5 |
| Calcium (mg/dL) | 10.1 | --- | --- | 7.8 |
| Sodium (mmol/L) | 160 | 154 | 154 | 150-165 |
| Potassium (mmol/L) | 4.7 | 4.6 | 3.5 | 3.5-5.8 |
| Sodium/potassium ratio | 34 | 33 | 44 | --- |
| Chloride (mmol/L) | 116 | **111** | **111** | 112-129 |
| Total protein (g/dL) | 8.2 | --- | 6.4 | 5.7-8.9 |
| Albumin (g/dL) | 3.5 | 3 | 2.4 | 2.2-4 |
| Globulins (g/dL) | 4.7 | --- | 4 | 2.8-5.1 |
| Albumin/globulins ratio | 0.7 | --- | 0.6 | --- |
| ALT (U/L) | 19 | 34 | 66 | 12-130 |
| ALP (U/L) | **<10** | 15 | **<10** | 14-111 |
| GGT (U/L) | 0 | --- | --- | 0-4 |
| Total bilirubin (mg/dL) | 0.4 | --- | --- | 0-0.9 |
| Cholesterol (mg/dL) | 180 | --- | --- | 65-225 |
| Osmolarity (mmol/kg) | 323 | --- | 306 | --- |
